# Adenosine Triphosphate (ATP) Is a Candidate Signaling Molecule in the Mitochondria-to-Nucleus Retrograde Response Pathway

**DOI:** 10.3390/genes4010086

**Published:** 2013-03-20

**Authors:** Feng Zhang, Tammy Pracheil, Janet Thornton, Zhengchang Liu

**Affiliations:** 1 Department of Molecular Biology, UT Southwestern Medical Center, Dallas, TX, USA; E-Mails: fengz@med.umich.edu (F.Z.); jlthornt26@hotmail.com (J.T.); 2 Department of Biological Sciences, University of New Orleans, 2000 Lakeshore Drive, New Orleans, LA 70148, USA; E-Mail: tmprache@my.uno.edu

**Keywords:** retrograde response, mitochondria to nucleus signaling, Rtg2, Mks1, ATP sensing, *Saccharomyces cerevisiae*, *K. lactis*, *K. waltii*

## Abstract

Intracellular communication from the mitochondria to the nucleus is achieved via the retrograde response. In budding yeast, the retrograde response, also known as the RTG pathway, is regulated positively by Rtg1, Rtg2, Rtg3 and Grr1 and negatively by Mks1, Lst8 and two 14-3-3 proteins, Bmh1/2. Activation of retrograde signaling leads to activation of Rtg1/3, two basic helix-loop-helix leucine zipper transcription factors. Rtg1/3 activation requires Rtg2, a cytoplasmic protein with an N-terminal adenosine triphosphate (ATP) binding domain belonging to the actin/Hsp70/sugar kinase superfamily. The critical regulatory step of the retrograde response is the interaction between Rtg2 and Mks1. Rtg2 binds to and inactivates Mks1, allowing for activation of Rtg1/3 and the RTG pathway. When the pathway is inactive, Mks1 has dissociated from Rtg2 and bound to Bmh1/2, preventing activation of Rtg1/3. What signals association or disassociation of Mks1 and Rtg2 is unknown. Here, we show that ATP at physiological concentrations dissociates Mks1 from Rtg2 in a highly cooperative fashion. We report that ATP-mediated dissociation of Mks1 from Rtg2 is conserved in two other fungal species, *K. lactis* and *K. waltii*. Activation of Rtg1/3 upregulates expression of genes encoding enzymes catalyzing the first three reactions of the Krebs cycle, which is coupled to ATP synthesis through oxidative phosphorylation. Therefore, we propose that the retrograde response is an ATP homeostasis pathway coupling ATP production with ATP-mediated repression of the retrograde response by releasing Mks1 from Rtg2.

## 1. Introduction

Mitochondria produce the majority of cells’ adenosine triphosphate (ATP) to be used as “energy currency” in eukaryotic cells. Apart from their metabolic function, mitochondria participate in a diverse array of physiological processes, such as apoptosis, cancer, degenerative diseases and aging [1,2,3,4]. Due to the versatility of mitochondrial functions, it's critically important for cells to monitor the functional state of mitochondria and adjust nuclear gene expression accordingly to achieve functional homeostasis of mitochondrial activity. This is achieved via the coordination of mitochondria-to-nucleus signaling pathways, known as the retrograde response [5,6]. The retrograde response adapts cells to changes in the functional state of mitochondria, such as respiratory defects, by mediating an assortment of cellular processes that include metabolic reconfiguration, nutrient sensing, aging and stress response pathways [7,8,9,10,11,12]. Retrograde response pathways have been reported in many eukaryotic organisms, including fungi, plants and animals [5,6,13,14,15,16]. In the budding yeast, *Saccharomyces cerevisiae*, the retrograde response, also known as the RTG pathway, mediates expression of many genes encoding proteins that function in small molecule transport, anaplerotic pathways, pleotropic drug resistance, aging and peroxisomal biogenesis [7,17,18,19,20,21,22].

The prototypal target gene of the RTG pathway is *CIT2*, encoding the peroxisomal isoform of citrate synthase [23,24]. In cells with reduced mitochondrial respiratory functions, *CIT2* expression is greatly induced, which requires three Rtg proteins, Rtg1, Rtg2 and Rtg3 [23,25]. Rtg1 and Rtg3 are two basic helix-loop-helix leucine zipper transcription factors that bind as a heterodimer to the promoter region of *CIT2* and activate *CIT2* expression [25]. Activation and nuclear translocation of Rtg1 and Rtg3 correlate with dephosphorylation of Rtg3 [26,27]. These processes require a novel cytoplasmic protein, Rtg2, which contains an N-terminal ATP binding domain of the Hsp70/acting/sugar kinase ATP binding domain superfamily [28,29,30]. The integrity of the ATP binding domain of Rtg2 is important for its interaction with Mks1 [28]. However, the underlying mechanism is still unknown. Activity of Rtg1 and Rtg3 can also be mediated by the target of the rapamycin (Tor) signaling pathway and the mitogen-activated protein kinase, Hog1, in the osmoregulatory signal transduction cascade, linking the retrograde response to other nutrient sensing and stress response pathways [28,31,32,33,34]. 

One main function of the RTG pathway is the biosynthesis of glutamate in cells with compromised respiratory functions [6]. Transcriptional regulation of the Krebs cycle genes, *CIT1*, *ACO1*, *IDH1* and *IDH2*, switches from the Hap2-5 complex to the Rtg1/3 complex in cells with respiratory deficiencies [18]. The products of these genes, as well as *CIT2* promote the synthesis of α-ketoglutarate, a precursor of glutamate. Mutations in *RTG* genes lead to glutamate auxotrophy in respiratory-deficient cells, underlying the role of the RTG pathway in glutamate homeostasis [18]. As a feedback control mechanism, glutamate is a potent repressor of the RTG pathway.

Activation of Rtg1 and Rtg3 by Rtg2 is indirect, and additional regulatory factors function between Rtg2 and Rtg1/3 [6]. These include a novel cytoplasmic protein, Mks1, Lst8 (a component of the Tor kinase complexes), Grr1 (a component of the SCF^Grr1^ E3 ubiquitin ligase) and two 14-3-3 proteins, Bmh1 and Bmh2 [28,32,33,35,36,37,38,39,40,41]. With the exception of Grr1, all of these factors are negative regulators of the RTG pathway. Among these proteins, Mks1 is a key regulatory component [6]. When active, Mks1 is dissociated from Rtg2, hyperphosphorylated and able to bind to Bmh1/2. Bmh1/2 binding prevents the SCF^Grr1^ E3 ubiquitin ligase-mediated ubiquitination and degradation of Mks1 [38]. It has been reported that Mks1 interacts with Tor1 and Tor2 kinases [42]. Since both Mks1 and Tor kinases are negative regulators of the RTG pathway, it is likely that the Mks1-Tor complex may directly phosphorylate and inactivate Rtg3. The role of Lst8 in the retrograde response pathway may also be linked to its role in the TOR kinase complexes.

The positive regulatory role of Rtg2 in the retrograde response is to bind to and inactivate Mks1. We have previously proposed that the interaction between Rtg2 and Mks1 constitutes a binary switch that turns the RTG pathway on or off [6,38]. A major unanswered question remains: What is the signaling molecule that mediates the interaction between Rtg2 and Mks1? Here, we present evidence to suggest that ATP is that signaling molecule. At physiological concentrations, ATP has an all-or-none effect on the interaction between Rtg2 and Mks1. We further show that ATP-dependent regulation of this interaction is evolutionarily conserved.

## 2. Experimental Section

### 2.1. Strains, Plasmids and Growth Media and Growth Conditions

Yeast strains and plasmids used in this study are listed in Table 1, Table 2, respectively. Yeast cells were grown in SD (0.67% yeast nitrogen base plus 2% dextrose), YNBcasD (SD medium plus 1% casamino acids) or YPD (1% yeast extract, 2% peptone, 2% dextrose) medium at 30 °C. When necessary, amino acids, adenine and/or uracil, were added to the growth medium at standard concentrations to cover auxotrophic requirements or at concentrations as indicated in the text and/or figure legends [43]. When needed, glutamate was added to the growth medium at the final concentration of 0.2% to inhibit the RTG pathway.

**Table 1:** Strains used in this study.

Strain	Genotype	Source	Application
RBY915	*MATα ura3-52 leu2 lys2 RTG2-myc3 mks1::LEU2*	[28]	Figure 1, Figure 2
TSY619	*MATα ura3-52 leu2 lys2 mks1::LEU2*	[38]	Figure 1
PSY142	*MATα ura3-52 leu2 lys2 ura3::CIT2-lacZ*	[41]	Figure 3, Figure 4
PSY142-rtg2	*MATα ura3-52 leu2 lys2 ura3::CIT2-lacZ rtg2::ura3*	[41]	Figure 3, Figure 4
ZLY145	*MATα ura3-52 leu2 lys2 ura3::CIT2-lacZ rtg2::ura3 mks1::kanMX4*	This study	Figure 4, Figure 5
ZLY028	*MATα ura3-52 leu2 lys2 ura3::CIT2-lacZ mks1::kanMX4*	[28]	Figure 4

**Table 2:** Plasmids used in this study.

Plasmid	Description	Source	Application
pZL1480	pRS416-MKS1p-MKS1-HA, expressing HA-tagged Mks1 from the *MKS1* promoter.	This study	Figure 1, Figure 2, Figure 4
pTS215	pRS416-MKS1, expressing *MKS1* from its own promoter.	[28]	Figure 1
pFZ142	pRS416-RTG2p-RTG2(Kla)-HA, expressing HA-tagged Rtg2 homolog from *K. lactis* from the *RTG2* promoter of *S. cerevisiae*.	This study	Figure 3
pFZ136	pRS416-RTG2p-RTG2(Kwa)-HA, expressing HA-tagged Rtg2 homolog from *K. waltii* from the *RTG2* promoter of *S. cerevisiae*.	This study	Figure 3
pZL927	pS416-MKS1, expressing Mks1 from the endogenous promoter.	[28]	Figure 4
pFZ138	pS416-MKS1p-MKS1(Kla), expressing the Mks1 homolog from *K. lactis* from the *MKS1* promoter of *S. cerevisiae*.	This study	Figure 4
pFZ144	pRS416-MKS1p-MKS1(Kwa), expressing the Mks1 homolog from *K. waltii* from the *MKS1* promoter of *S. cerevisiae*.	This study	Figure 4
pFZ134	pRS416-MKS1p-MKS1(Kla)-HA3, expressing HA-tagged Mks1 homolog from *K. lactis* from the *MKS1* promoter of *S. cerevisiae*.	This study	Figure 4
pFL150	pRS416-MKS1p-MKS1(Kwa)-HA3, expressing HA-tagged Mks1 homolog from *K. waltii* from the *MKS1* promoter of *S. cerevisiae*.	This study	Figure 4
pZL1951	pRS415-RTG2-myc, expressing myc-tagged Rtg2 from the *RTG2* promoter.	This study	Figure 5
pFZ140	pRS415-RTG2p-RTG2(Kla)-myc, expressing myc-tagged Rtg2 homolog from *K. lactis* from the *RTG2* promoter of *S. cerevisiae*.	This study	Figure 5
pFZ148	pRS415-RTG2p-RTG2(Kwa)-myc, expressing myc-tagged Rtg2 homolog from *K. waltii* from the *RTG2* promoter of *S. cerevisiae*.	This study	Figure 5

### 2.2. Cellular Extracts Preparation, Immunoprecipitation and Immunoblotting

Total cellular protein extracts were prepared by using the NaOH-β-mercaptoethanol method as described [44]. For interaction assays between Rtg2 and Mks1 in the presence of ATP or other nucleotides, cellular lysates were prepared in IP buffer (20 mM Hepes-KOH pH 7.6, 150 mM KCl, 10 mM MgCl, 0.5% Triton X-100 and protease inhibitors). Cell extracts (~1.6 mg proteins) were incubated at 4 °C with ATP or other nucleotides as indicated for 1.5 h, after which 2 μg anti-myc antibody (9E10, Roche) was added and incubated for 1.5 h. Forty microliters 50% slurry of protein G-Sepharose (Roche) was then added to each sample, and the samples were further incubated at 4 °C for 2 h. Immunoprecipitates were washed five times each with 1 ml IP buffer. Proteins bound to the Sepharose beads were released by boiling in 1X SDS-PAGE loading buffer. The released immune complexes were analyzed by SDS-PAGE and immunoblotting. myc and HA-tagged proteins were probed with anti-myc antibody 9E10 and anti-HA antibody 12CA5, respectively. To determine the effect of nucleotides on the dissociation of Rtg2 and Mks1, immunoprecipitates of Rtg2-myc and Mks1-HA were prepared using the procedure mentioned above, with the exception that ATP was not used. The immunopurified Rtg2-Mks1 complex was then incubated in the presence of various nucleotides at indicated concentrations for 5 minutes at 23 °C, after which the supernatant and pellet fractions were obtained by 2 min. of centrifugation. Rtg2-myc and Mks1-HA in the supernatant and pellet fractions were separated by SDS-PAGE and detected by immunoblotting. 

### 2.3. Yeast Transformation and β-Galactosidase Activity Assays

Plasmids were transformed into yeast strains using the high-efficiency lithium acetate-PEG method [43]. β-galactosidase assays were carried out as described [43]. For each plasmid and strain combination, assays were conducted in triplicate, and independent experiments were carried out two or three times.

## 3. Results and Discussion

### 3.1. ATP, but not ADP or AMP-PNP, at High Concentrations Disrupts the Interaction between Rtg2 and Mks1 in Total Cellular Lysates

**Figure 1:**
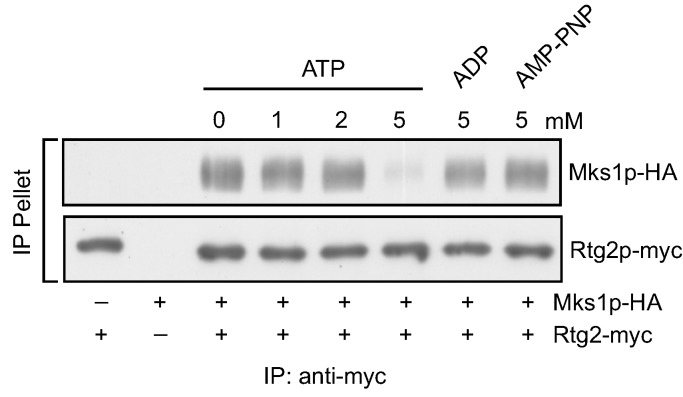
Adenosine triphosphate (ATP) disrupts the interaction between Rtg2 and Mks1 in total cellular lysates. Cellular lysates prepared from yeast cells expressing Rtg2-myc and/or Mks1-HA as indicated were analyzed for the effect of ATP, ADP and adenosine 5′-(β,γ-imido) triphosphate (AMP-PNP) on the interaction between Rtg2 and Mks1 by immunoprecipitation, as described in the Experimental Section. Cellular lysates were treated with the nucleotides at indicated concentrations, and Rtg2-myc was immunoprecipitated using anti-myc anti-body and protein G Sepharose beads. Rtg2-myc and Mks1-HA in IP pellet fractions were detected by immunoblotting.

Retrograde signaling is regulated by the dynamic interaction between Rtg2 and Mks1. When the RTG pathway is inactive, Mks1 is dissociated from Rtg2 and inhibits Rtg1/3. When the RTG pathway is active, Mks1 is sequestered by Rtg2. To understand the mechanism by which this interaction is regulated, we tested candidate molecules that may mediate the interaction between Rtg2 and Mks1. Since Rtg2 has an N-terminal ATP binding domain and ATP is produced by mitochondria through oxidative phosphorylation, one possible candidate signaling molecule is ATP. We hypothesized that ATP production from robust mitochondrial respiratory metabolism releases Mks1 from Rtg2 to inhibit the RTG pathway, which may explain why the RTG pathway is not active in cells with robust respiration. Accordingly, we tested whether ATP affects the interaction between Rtg2 and Mks1. We prepared cellular extracts from cells co-expressing functional myc-tagged Rtg2 and HA-tagged Mks1 and incubated with different concentrations of ATP. We then immunoprecipitated Rtg2-myc with anti-myc anti-body immobilized on protein G Sepharose beads and determined the amount of Mks1-HA in the Rtg2-myc immunoprecipitates. Figure 1 shows that without the addition of exogenous ATP, Mks1-HA was efficiently pulled down with Rtg2-myc. Similarly, treatment with 1 and 2 mM ATP had little or no effect on the interaction between Rtg2 and Mks1. Interestingly, treatment with 5 mM ATP greatly reduced the amount of Mks1-HA recovered in the Rtg2-myc immunoprecipitates. In contrast, treatment with 5 mM ADP had little effect on the interaction between Rtg2 and Mks1. We also treated total cellular extracts with 5 mM adenosine 5′-(β,γ-imido) triphosphate (AMP-PNP), which is a non-hydrolysable analogue of ATP, and found that it also had no effect on the interaction between Rtg2-myc and Mks1-HA, suggesting that ATP hydrolysis may be required for the release of Mks1 from Rtg2. 

### 3.2. ATP within a Small Range of Physiological Concentrations Releases Mks1 from an Immunopurified Rtg2-Mks1 Complex

**Figure 2:**
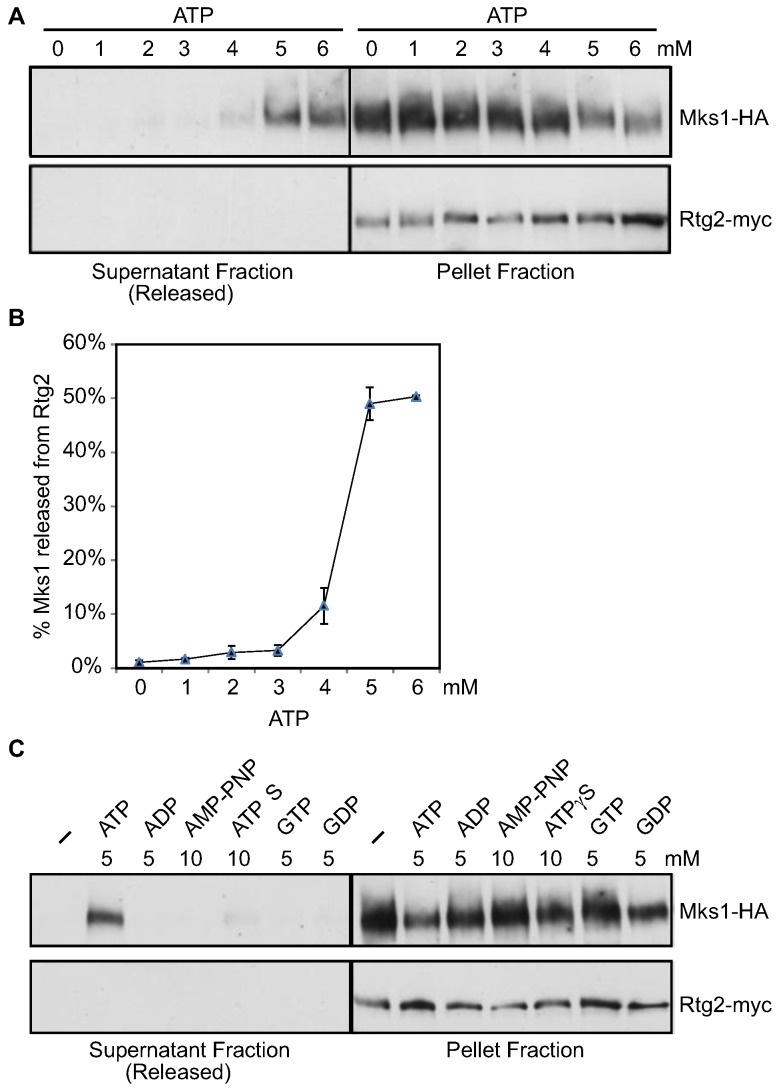
The effect of ATP titration on Mks1 release from Rtg2. (**A**) Immunopurified Rtg2-myc-Mks1-HA complex from RBY915 cells co-expressing Rtg2-myc and Mks1-HA was incubated with titrating levels of ATP, and the presence of Rtg2-myc and Mks1-HA in the pellet and supernatant (released) fractions were determined by Western-blotting. (**B**) Quantitative analysis of the amount of Mks1 released from Rtg2 in the presence of ATP. The result was the average of two independent experiments. (**C**) The effect of 5 mM ADP, GTP or GDP and 10 mM AMP-PNP or ATPγS on the interaction between Rtg2 and Mks1.

Rtg2 has a predicted N-terminal ATP binding domain, the integrity of which is important for the function of Rtg2 [28,29]. Data in Figure 1 suggest that ATP may directly bind to Rtg2, resulting in Mks1 release. Before testing this possibility, we considered that total cellular lysates contain several thousand proteins, potentially complicating interaction analysis between Mks1 and Rtg2. Therefore, using aliquots of an immunopurified complex, we tested the effect of ATP at various concentrations on the interaction between Rtg2 and Mks1. In preliminary experiments, we found that 5 mM ATP can lead to maximum release of Mks1 from Rtg2, and a further increase to up to 10 mM did not result in the release of more Mks1 from Rtg2 (data not shown). Therefore, we treated the Rtg2-Mks1 complex with 0~6 mM ATP at 1 mM intervals. Figure 2A shows that while treatment with 1~3 mM ATP had little or no effect on the interaction between Rtg2 and Mks1, 5 and 6 mM ATP treatment efficiently released Mks1-HA from Rtg2-Mks1, and 4 mM ATP had an intermediate effect. Quantitative analysis of data in Figure 2A shows that ~50% Mks1 is released from Rtg2 in the presence of 5~6 mM ATP (Figure 2B). The appearance of Mks1 in the supernatant fraction was not due to the release of Rtg2-myc from anti-myc antibody immobilized on the protein G Sepharose beads, since Rtg2-myc was not detected in the supernatant fraction of samples treated with different concentrations of ATP. Next, we examined the effect of three other purine nucleotides, ADP, GTP and GDP, and two ATP analogs, AMP-PNP and 5'-O-(3-thio) triphosphate (ATPγS), on the interaction between Rtg2 and Mks1. We found that at 5 mM concentration, ADP, GTP or GDP had no effect. At 10 mM concentration, the non-hydrolysable ATP analog, AMP-PNP, also had no effect. Interestingly, at 10 mM concentration, ATPγS, a slowly hydrolysable ATP analog [45,46], could weakly dissociate Mks1 from Rtg2. Together, this data indicate that Mks1 release from Rtg2 by ATP is specific and that ATP hydrolysis is required for this process. 

We attempted to purify recombinant Rtg2 and Mks1 to determine whether these two proteins alone are sufficient to recapitulate the ATP effect observed with immunopurified complex. However, numerous attempts made to purify recombinant Rtg2 from bacterial, yeast and insect cells failed, due to its insolubility. Therefore, the possibility that other proteins in the immunopurified Rtg2-Mks1 complex contribute to their interaction or dissociation upon ATP treatment cannot be ruled out.

Intracellular ATP concentration is estimated to be in the range of 1–5 mM [47,48]. The data in Figure 2 present two striking observations: ATP has an all-or-none effect on the interaction between Rtg2 in Mks1; the concentration of ATP that dramatically changes the interaction between Rtg2 and Mks1 is within the physiological range. Since activation of the RTG pathway leads to increased expression of genes encoding citrate synthase, aconitase and isocitrate dehydrogenase, increased activities of these enzymes are expected to lead to increased metabolic flux into the Krebs cycle and ATP production in mitochondria. Our data in Figure 2 shows that ATP dissociates Mks1 from Rtg2, which results in inhibition of the RTG pathway. Taken together, we propose that ATP is the mitochondria-derived signaling molecule that turns off the pathway to achieve ATP homeostasis. ATP is the universal energy currency in biological systems; therefore, if our hypothesis is correct, we expect to find that the interaction between Mks1 and Rtg2 homologs in other fungal species is similarly regulated by ATP.

### 3.3. RTG2 Homologs from *K. lactis* and *K. waltii* Complement an rtg2Δ Mutation in *S. cerevisiae*

Two fungal species, *K. lactis* and *K. waltii*, contain both Rtg2 and Mks1 homologs [49]. If the retrograde response pathway in budding yeast mediates ATP homeostasis, we expect the effect of ATP on the release of Mks1 from Rtg2 to be conserved in other fungal species. To test this possibility, we first cloned the *RTG2* homologs from *K. lactis* and *K. waltii* and determined whether they complement an *rtg2Δ* mutation in *S. cerevisiae*.

**Figure 3:**
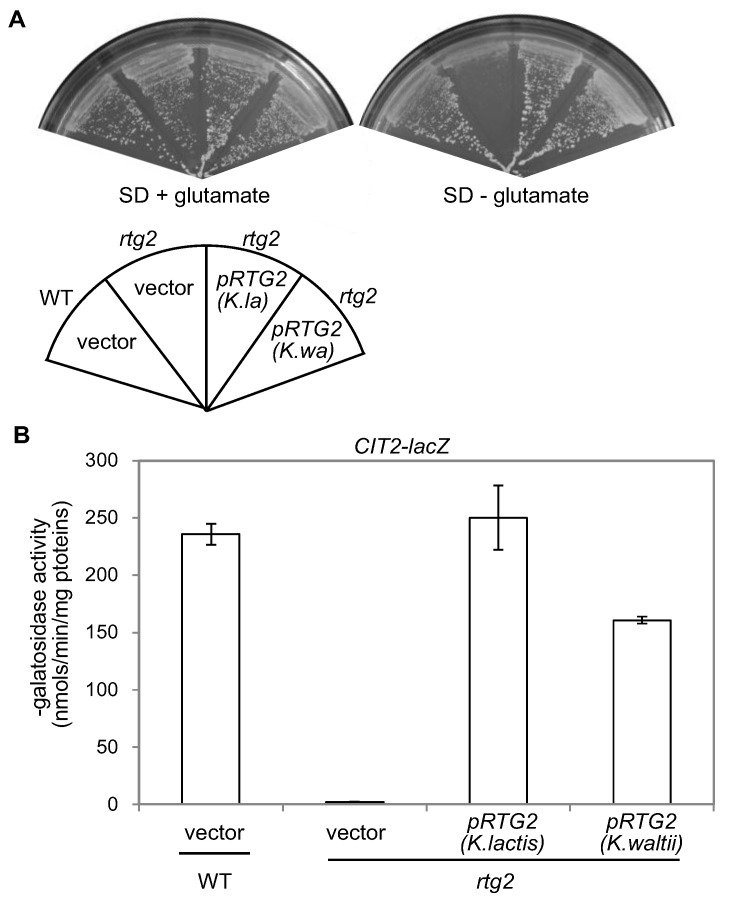
The Rtg2 homologs from *K. lactis* and *K. waltii* are functional in *S. cerevisiae.* (**A**) *RTG2* homologs complement glutamate auxotrophy phenotype of *rtg2Δ* in *S. cerevisiae.* Wild-type (WT, PSY142) and *rtg2Δ* (PSY142-rtg2) mutant cells carrying empty vector or centromeric plasmids encoding *RTG2* genes from *K. lactis* (*K.la*) and *K. waltii* (*K.wa*) were grown on SD medium with or without glutamate at 30 °C for 2–3 days. (**B**) Expression of *RTG2* homologs restores expression of a *CIT2-lacZ* reporter to *rtg2Δ* mutant cells in *S cerevisiae*. Yeast strains described for panel (A) were grown in YNBcasD medium to mid-log phase, and β-galactosidase assays were conducted as described in the Experimental Section.

The Rtg2 homolog in *K. lactis* and in *K. waltii* is a 587 and 583-residue protein, respectively. Both proteins show ~69% sequence identity to *S. cerevisiae* Rtg2 (data not shown). As reported previously, the RTG pathway is required for the biosynthesis of α-ketoglutarate, the precursor to glutamate, and an *rtg2Δ* mutant strain is unable to grow on minimal SD medium without glutamate (Figure 3A) [18,23]. Expression of the Rtg2 homolog from both *K. lactis* and *K. waltii* under the control of the *S. cerevisiae* *RTG2* promoter enabled *rtg2Δ* mutant cells to grow on SD medium without glutamate (Figure 3A). Expression of *CIT2*, encoding the peroxisomal isoform of citrate synthase, requires Rtg2 [23]. Figure 3B shows that expression of Rtg2 homologs from *K. lactis* and *K. waltii* restored expression of *CIT2-lacZ* in *rtg2Δ* mutant cells to near wild-type levels. Together, these data suggest that the function of Rtg2 is conserved. 

### 3.4. MKS1 Homologs from *K. lactis* and *K. waltii* Complement an mks1Δ Mutation in *S. cerevisiae*

**Figure 4:**
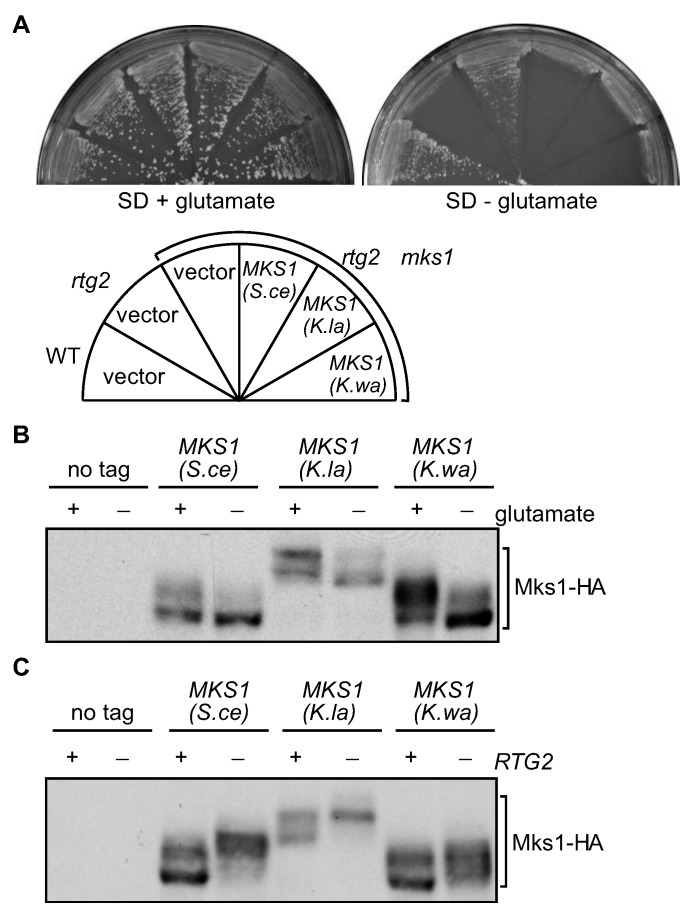
The function and regulation of Mks1 homologs from *K. lactis* and *K. waltii* are conserved. **(A)** *MKS1* homologs from *K. lactis* (*K.la*) and *K. waltii* (*K.wa*) complement an *mks1Δ* mutation in *S. cerevisiae* (*S.ce*)*.* Yeast strains as indicated were grown on SD medium with or without glutamate at 30 °C for 2 to 3 days. (**B**) Glutamate has similar effects on the phosphorylation of *S. cerevisiae* Mks1 and its homologs from *K. lactis* and *K. waltii*. *mks1Δ* mutant cells (ZLY028) carrying centromeric plasmids encoding *MKS1* genes from the indicated fungal species were grown in SD medium supplemented with or without glutamate. Total cellular proteins were prepared and separated by SDS-PAGE, and HA-tagged Mks1 was detected by Western-blotting. (**C**) The absence of *RTG2* increases phosphorylation of Mks1. Cells expressing HA-tagged Mks1 from the indicated fungal species without (*+ RTG2*) or with an *rtg2Δ* mutation (˗ *RTG2*) were grown in YNBcasD medium, and phosphorylation of Mks1 was analyzed as described for panel (B).

The Mks1 homolog in *K. lactis* and *K. waltii* is a 638 and 588-residue protein, respectively. Unlike Rtg2 proteins, Mks1 proteins are much less conserved: *S. cerevisiae* Mks1 shares ~29% sequence identity with its homologs in *K. lactis* and *K. waltii* (data not shown). We tested whether Mks1 homologs could complement an *mks1Δ* mutation in *S. cerevisiae*. Mks1 is a negative regulator of the RTG pathway, and an *mks1Δ* mutation bypasses the requirement of Rtg2 for cells to grow on medium without glutamate [33,35]. Therefore, we generated plasmids encoding *MKS1* homologs under the control of the *MKS1* promoter from *S. cerevisiae* and transformed them into *rtg2Δ* *mks1Δ* double mutant cells. Figure 4A shows that expression of the Mks1 homologs in *rtg2Δ* *mks1Δ* double mutant cells resulted in glutamate auxotrophy, indicating that Mks1 homologs from *K. lactis* and *K. waltii* are functional in *S. cerevisiae*.

Mks1 is a phosphoprotein, whose phosphorylation correlates with its activity in cells: when it is bound to Rtg2 and inactive, Mks1 is hypophosphorylated; when the RTG pathway is inactive, Mks1 becomes hyperphosphorylated [33,35]. We then determined whether the regulation of Mks1 is also conserved. Figure 4B shows that in the presence of glutamate, which inhibits the retrograde pathway, Mks1 proteins from *S. cerevisiae*, *K. lactis* and *K. waltii*, all became hyperphosphorylated. Mks1 phosphorylation in *S. cerevisiae* is also dependent on the availability of Rtg2: in the absence of Rtg2, Mks1 becomes hyperphosphorylated (Figure 4C) [33,35]. We found that the Mks1 homologs from *K. lactis* and *K. waltii* were also hyperphosphorylated in *rtg2Δ* mutant cells (Figure 4C). Together, these data suggest that the function and regulation of Mks1 proteins are conserved. 

### 3.5. ATP at Physiological Concentrations Releases Mks1 Homologs from its Cognate Rtg2 Homologs from *K. lactis* and *K. waltii*

**Figure 5:**
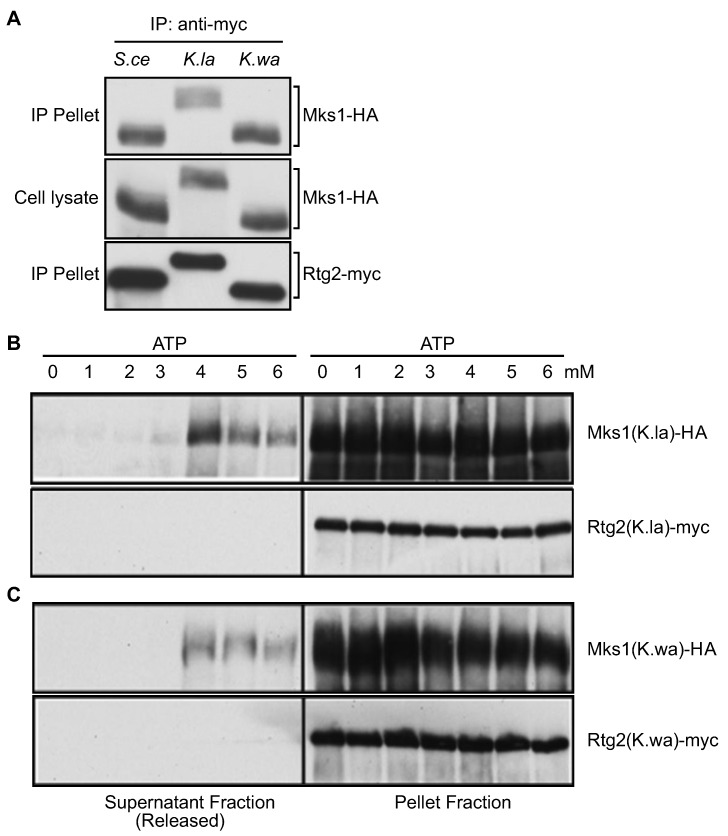
ATP releases Mks1 from Rtg2. (**A**) Rtg2 and Mks1 homologs from *K. lactis* and *K. waltii* form a complex. *rtg2Δ* *mks1Δ* double mutant cells (ZLY145) expressing pairs of epitope-tagged Rtg2 and Mks1 from indicated fungal species were analyzed for interaction between Rtg2 and Mks1 using co-immunoprecipitation. Rtg2-myc was precipitated using anti-myc anti-body. Rtg2-myc and Mks1-HA were detected by immunoblotting. (**B** and **C**) ATP at physiological concentrations has an all-or-none effect on releasing the Mks1 homolog from the Rtg2 homolog from *K. lactis* (B) and *K. waltii* (C). The effect of ATP on the release of Mks1 from immunopurified Rtg2-Mks1 complexes was analyzed as described for Figure 2.

After confirming that Rtg2 and Mks1 homologs from *K. lactis* and *K. waltii* are functional, we then looked at whether interactions between Rtg2 and Mks1 homologs are regulated by physiological concentrations of ATP. Figure 5A shows that when expressed in *S. cerevisiae* *rtg2Δ mks1Δ* double mutant cells, pairs of Mks1 and Rtg2 from *S. cerevisiae*, *K. lactis* and *K waltii* form a complex using co-immunoprecipitation. We then immunopurified *K. lactis* and *K. waltii* Rtg2-Mks1 complexes and treated them with ATP at different concentrations. Figure 5 B and C show that 1–3 mM ATP had little or no effect on Mks1 release from Rtg2. Treatment with 4 mM ATP resulted in maximal release of Mks1 from Rtg2. Similar effects of physiological levels of ATP on the interaction of Rtg2 and Mks1 in three different fungal species strongly suggest that the RTG pathway mediates ATP homeostasis. 

Cellular energy homeostasis has been proposed to be mediated by AMP-activated protein kinase (AMPK) in eukaryotes [50,51]. AMPK is activated by a rise in the AMP:ATP ratio. When cellular energy levels drop, AMP levels rise, which leads to the activation of AMPK. Once activated, AMPK turns on catabolic pathways that generate ATP and turns off processes that utilize ATP. The *S. cerevisiae* AMPK, Snf1, is required for cell growth on less preferred fermentable and non-fermentable carbon sources, conditions that often require robust mitochondrial respiratory function [52,53]. Since the RTG pathway is more active in respiratory deficient cells, we propose that the RTG pathway and AMPK may regulate energy homeostasis under different conditions. It is also possible that these two may work together to achieve finer control of cellular energy levels.

## 4. Conclusions

**Figure 6:**
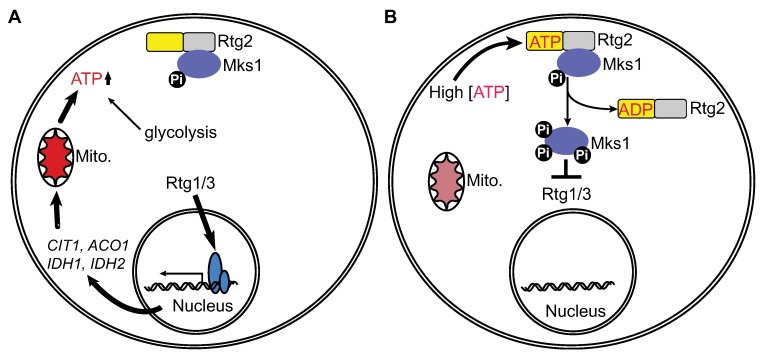
A model for the role of ATP-mediated interaction between Rtg2 and Mks1 in ATP homeostasis. See text for details.

The retrograde response senses changes in the functional state of mitochondria and adjusts nuclear gene expression accordingly. The signaling molecule linking the functional state of the mitochondria to the RTG pathway has been elusive. Here, we provide evidence that ATP may be this long sought-after signaling molecule. A key regulatory step of this pathway is the interaction between Rtg2 and Mks1. We find that ATP has an all-or-none effect on releasing Mks1 from Rtg2 in three different fungal species. Furthermore, the concentration of ATP that elicits the all-or-none effect is within the physiological range of ATP. We report that Rtg2 and Mks1 homologs from two other fungal species, *K. lactis* and *K. waltii*, are able to complement *rtg2Δ* and *mks1Δ* mutations in *S. cerevisiae*, respectively. Rtg2 and Mks1 homologs from *K. lactis* and *K. waltii* can form a complex, and their interaction is similarly regulated by ATP. Here, we propose that the retrograde response mediates ATP homeostasis by participating in a conserved negative feedback loop that responds to ATP levels to shut off ATP production when ATP is in excess: the RTG pathway regulates the expression of genes encoding the first three Krebs cycle enzymes, and activation of this pathway is expected to increase the metabolic flux into the Krebs cycle and ATP synthesis in mitochondria (Figure 6A). When the level of cellular ATP reaches a certain threshold (3–4.5 mM), ATP releases Mks1 from Rtg2 to turn off the RTG pathway (Figure 6B). Together, these two processes help achieve cellular ATP homeostasis. 

## References

[1] Jiang X., Wang X. (2004). Cytochrome C-mediated apoptosis. Annu. Rev. Biochem..

[2] Orrenius S., Gogvadze V., Zhivotovsky B. (2007). Mitochondrial oxidative stress: Implications for cell death. Annu. Rev. Pharmacol. Toxicol..

[3] Seo A.Y., Joseph A.M., Dutta D., Hwang J.C., Aris J.P., Leeuwenburgh C. (2010). New insights into the role of mitochondria in aging: Mitochondrial dynamics and more. J. Cell. Sci..

[4] Wallace D.C. (2005). A mitochondrial paradigm of metabolic and degenerative diseases, aging, and cancer: A dawn for evolutionary medicine. Annu. Rev. Genet..

[5] Butow R.A., Avadhani N.G. (2004). Mitochondrial signaling: the retrograde response. Mol. Cell..

[6] Liu Z., Butow R.A. (2006). Mitochondrial retrograde signaling. Annu. Rev. Genet..

[7] Epstein C.B., Waddle J.A., Hale W.T., Dave V., Thornton J., Macatee T.L., Garner H.R., Butow R.A. (2001). Genome-wide responses to mitochondrial dysfunction. Mol. Biol. Cell..

[8] Biswas G., Anandatheerthavarada H.K., Zaidi M., Avadhani N.G. (2003). Mitochondria to nucleus stress signaling: A distinctive mechanism of NFkappaB/Rel activation through calcineurin-mediated inactivation of IkappaBbeta. J. Cell. Biol..

[9] Biswas G., Guha M., Avadhani N.G. (2005). Mitochondria-to-nucleus stress signaling in mammalian cells: Nature of nuclear gene targets, transcription regulation, and induced resistance to apoptosis. Gene.

[10] Guha M., Fang J.K., Monks R., Birnbaum M.J., Avadhani N.G. (2010). Activation of Akt is essential for the propagation of mitochondrial respiratory stress signaling and activation of the transcriptional coactivator heterogeneous ribonucleoprotein A2. Mol. Biol. Cell..

[11] Guha M., Tang W., Sondheimer N., Avadhani N.G. (2010). Role of calcineurin, hnRNPA2 and Akt in mitochondrial respiratory stress-mediated transcription activation of nuclear gene targets. Biochim. Biophys Acta.

[12] Jazwinski S.M., Kriete A. (2012). The yeast retrograde response as a model of intracellular signaling of mitochondrial dysfunction. Front. Physiol..

[13] Dojcinovic D., Krosting J., Harris A.J., Wagner D.J., Rhoads D.M. (2005). Identification of a region of the Arabidopsis AtAOX1a promoter necessary for mitochondrial retrograde regulation of expression. Plant. Mol. Biol..

[14] Scheckhuber C.Q., Houthoofd K., Weil A.C., Werner A., De Vreese A., Vanfleteren J.R., Osiewacz H.D. (2011). Alternative oxidase dependent respiration leads to an increased mitochondrial content in two long-lived mutants of the aging model Podospora anserina. PLoS One.

[15] Rhoads D.M., Subbaiah C.C. (2007). Mitochondrial retrograde regulation in plants. Mitochondrion.

[16] Yang J., Zhang M., Yu J. (2008). Mitochondrial retrograde regulation tuning fork in nuclear genes expressions of higher plants. J. Genet. Genomics.

[17] Traven A., Wong J.M., Xu D., Sopta M., Ingles C.J. (2001). Interorganellar communication. Altered nuclear gene expression profiles in a yeast mitochondrial dna mutant. J. Biol. Chem..

[18] Liu Z., Butow R.A. (1999). A transcriptional switch in the expression of yeast tricarboxylic acid cycle genes in response to a reduction or loss of respiratory function. Mol. Cell. Biol..

[19] Moye-Rowley W.S. (2005). Retrograde regulation of multidrug resistance in Saccharomyces cerevisiae. Gene.

[20] McCammon M.T., Epstein C.B., Przybyla-Zawislak B., McAlister-Henn L., Butow R.A. (2003). Global transcription analysis of Krebs tricarboxylic acid cycle mutants reveals an alternating pattern of gene expression and effects on hypoxic and oxidative genes. Mol. Biol. Cell..

[21] Woo D.K., Poyton R.O. (2009). The absence of a mitochondrial genome in rho0 yeast cells extends lifespan independently of retrograde regulation. Exp. Gerontology.

[22] Miceli M.V., Jiang J.C., Tiwari A., Rodriguez-Quinones J.F., Jazwinski S.M. (2011). Loss of mitochondrial membrane potential triggers the retrograde response extending yeast replicative lifespan. Front. Genet..

[23] Liao X., Butow R.A. (1993). RTG1 and RTG2: two yeast genes required for a novel path of communication from mitochondria to the nucleus. Cell.

[24] Liao X.S., Small W.C., Srere P.A., Butow R.A. (1991). Intramitochondrial functions regulate nonmitochondrial citrate synthase (CIT2) expression in Saccharomyces cerevisiae. Mol. Cell. Biol..

[25] Jia Y., Rothermel B., Thornton J., Butow R.A. (1997). A basic helix-loop-helix-leucine zipper transcription complex in yeast functions in a signaling pathway from mitochondria to the nucleus. Mol. Cell. Biol..

[26] Sekito T., Thornton J., Butow R.A. (2000). Mitochondria-to-nuclear signaling is regulated by the subcellular localization of the transcription factors Rtg1p and Rtg3p. Mol. Biol. Cell..

[27] Dilova I., Powers T. (2006). Accounting for strain-specific differences during RTG target gene regulation in Saccharomyces cerevisiae. FEMS Yeast Res..

[28] Liu Z., Sekito T., Spirek M., Thornton J., Butow R.A. (2003). Retrograde signaling is regulated by the dynamic interaction between Rtg2p and Mks1p. Mol. Cell..

[29] Koonin E.V. (1994). Yeast protein controlling inter-organelle communication is related to bacterial phosphatases containing the Hsp 70-type ATP-binding domain. Trends Biochem. Sci..

[30] Bork P., Sander C., Valencia A. (1992). An ATPase domain common to prokaryotic cell cycle proteins, sugar kinases, actin, and hsp70 heat shock proteins. Proc. Natl. Acad. Sci USA.

[31] Ruiz-Roig C., Noriega N., Duch A., Posas F., de Nadal E. (2012). The Hog1 SAPK controls the Rtg1/Rtg3 transcriptional complex activity by multiple regulatory mechanisms. Mol. Biol. Cell..

[32] Dilova I., Aronova S., Chen J.C., Powers T. (2004). Tor signaling and nutrient-based signals converge on Mks1p phosphorylation to regulate expression of Rtg1.Rtg3p-dependent target genes. J. Biol. Chem..

[33] Dilova I., Chen C.Y., Powers T. (2002). Mks1 in concert with TOR signaling negatively regulates RTG target gene expression in S. cerevisiae. Curr. Biol..

[34] Komeili A., Wedaman K.P., O'Shea E.K., Powers T. (2000). Mechanism of metabolic control. Target of rapamycin signaling links nitrogen quality to the activity of the Rtg1 and Rtg3 transcription factors. J. Cell. Biol..

[35] Sekito T., Liu Z., Thornton J., Butow R.A. (2002). RTG-dependent mitochondria-to-nucleus signaling is regulated by MKS1 and is linked to formation of yeast prion [URE3]. Mol. Biol. Cell..

[36] Tate J.J., Cox K.H., Rai R., Cooper T.G. (2002). Mks1p is required for negative regulation of retrograde gene expression in Saccharomyces cerevisiae but does not affect nitrogen catabolite repression-sensitive gene expression. J. Biol. Chem..

[37] Ferreira Junior J.R., Spirek M., Liu Z., Butow R.A. (2005). Interaction between Rtg2p and Mks1p in the regulation of the RTG pathway of Saccharomyces cerevisiae. Gene.

[38] Liu Z., Spirek M., Thornton J., Butow R.A. (2005). A novel degron-mediated degradation of the RTG pathway regulator, Mks1p, by SCFGrr1. Mol. Biol. Cell..

[39] Chen E.J., Kaiser C.A. (2003). LST8 negatively regulates amino acid biosynthesis as a component of the TOR pathway. J. Cell. Biol..

[40] Chen E.J., Kaiser C.A. (2002). Amino acids regulate the intracellular trafficking of the general amino acid permease of Saccharomycescerevisiae. Proc. Natl. Acad. Sci. USA.

[41] Liu Z., Sekito T., Epstein C.B., Butow R.A. (2001). RTG-dependent mitochondria to nucleus signaling is negatively regulated by the seven WD-repeat protein Lst8p. Embo. J..

[42] Breitkreutz A., Choi H., Sharom J.R., Boucher L., Neduva V., Larsen B., Lin Z.Y., Breitkreutz B.J., Stark C., Liu G., Ahn J., Dewar-Darch D., Reguly T., Tang X., Almeida R., Qin Z.S., Pawson T., Gingras A.C., Nesvizhskii A.I., Tyers M. (2010). A global protein kinase and phosphatase interaction network in yeast. Science.

[43] Amberg D.C., Burke D.J., Strathern J.N. (2005). Methods in Yeast Genetics: A Cold Spring Harbor Laboratory Course Manual.

[44] Yaffe M.P., Schatz G. (1984). Two nuclear mutations that block mitochondrial protein import in yeast. Proc. Natl. Acad. Sci. USA.

[45] Paulus B.F., Bryant F.R. (1997). Time-dependent inhibition of recA protein-catalyzed ATP hydrolysis by ATPgammaS: Evidence for a rate-determining isomerization of the recA-ssDNA complex. Biochemistry.

[46] Yu X., Egelman E.H. (1992). Direct visualization of dynamics and co-operative conformational changes within RecA filaments that appear to be associated with the hydrolysis of adenosine 5'-O-(3-thiotriphosphate). J. Mol. Biol..

[47] Larsson C., Nilsson A., Blomberg A., Gustafsson L. (1997). Glycolytic flux is conditionally correlated with ATP concentration in Saccharomyces cerevisiae: A chemostat study under carbon- or nitrogen-limiting conditions. J. Bacteriol..

[48] Sauer U., Schlattner U. (2004). Inverse metabolic engineering with phosphagen kinase systems improves the cellular energy state. Metab. Eng..

[49] Liu Z., Butow R.A. (2006). Mitochondrial Retrograde Signaling. Annu. Rev. Genet..

[50] Hardie D.G. (2011). AMP-activated protein kinase: An energy sensor that regulates all aspects of cell function. Genes Dev..

[51] Carling D., Thornton C., Woods A., Sanders M.J. (2012). AMP-activated protein kinase: New regulation, new roles?. Biochem. J..

[52] Schuller H.J. (2003). Transcriptional control of nonfermentative metabolism in the yeast Saccharomyces cerevisiae. Curr. Genet..

[53] Zaman S., Lippman S.I., Zhao X., Broach J.R. (2008). How Saccharomyces responds to nutrients. Annu. Rev. Genet..

